# The effect of test modality on dynamic exercise biomarkers in children, adolescents, and young adults

**DOI:** 10.14814/phy2.14178

**Published:** 2019-07-28

**Authors:** Ronen Bar‐Yoseph, Janos Porszasz, Shlomit Radom‐Aizik, Kim D. Lu, Annamarie Stehli, Pearl Law, Dan M. Cooper

**Affiliations:** ^1^ Pediatric Exercise and Genomics Research Center (PERC), Department of Pediatrics University of California Irvine Irvine California; ^2^ Rehabilitation Clinical Trials Center, Division of Respiratory and Critical Care Physiology and Medicine Los Angeles Biomedical Research Institute at Harbor‐UCLA Medical Center Torrance California; ^3^ University of California Irvine Institute for Clinical and Translational Science Irvine California

**Keywords:** Cycle ergometry, peak oxygen uptake ($\dot{V}$), puberty, submaximal biomarkers of fitness, treadmill

## Abstract

Cardiopulmonary exercise testing (CPET) modalities, treadmill (TM), and cycle ergometer (CE), influence maximal gas exchange and heart rate (HR) responses. Little is known regarding CPET modality effect on submaximal biomarkers during childhood and adolescence. Ninety‐four healthy participants (7–34 y.o., 53% female) performed TM and CE CPET to address two major gaps: (1) the effect of modality on submaximal CPET biomarkers, and (2) estimation of work rate in TM CPET. Breath‐by‐breath gas exchange enabled calculation of linear regression slopes such as $\dot{V}$O_2_/ΔHR and Δ$\dot{V}$E/Δ$\dot{V}$CO_2_. Lean body mass (LBM) was measured with dual X‐ray absorptiometry. We tested a novel TM CPET estimate of work rate based on TM velocity^2^, incline, and body mass (VIM). Like the linear relationship between $\dot{V}$O_2_ and work rate in CE CPET, $\dot{V}$O_2_ increased linearly with TM VIM. TM Δ$\dot{V}$O_2_/ΔHR was highly correlated with CE (*r* = 0.92), and each increased substantially with LBM (*P* < 0.0001 for TM and CE). Δ$\dot{V}$O_2_/ΔHR was to a small (~8.7%) but significant extent larger in TM (1.6 mL/min/beat, *P* = 0.04). In contrast, TM and CE Δ$\dot{V}$E/Δ$\dot{V}$CO_2_ decreased significantly with LBM, supporting earlier observations from CE CPET. For both CE and TM, males had significantly higher Δ$\dot{V}$O_2_/ΔHR but lower Δ$\dot{V}$E/Δ$\dot{V}$CO_2_ than females. Novel TM CPET biomarkers such as ΔVIM/ΔHR and ∆$\dot{V}$O_2_/ΔVIM paralleled effects of LBM observed in CE CPET. TM and CE CPET submaximal biomarkers are not interchangeable, but similarly reflect maturation during critical periods. CPET analysis that utilizes data actually measured (rather than estimated) may improve the clinical value of TM and CE CPET.

## Introduction

The goal of this study was to test hypotheses focused on the effect of the two most common exercise testing modalities, cycle ergometry (CE), and treadmill (TM), on cardiopulmonary exercise testing (CPET) results in children, adolescents, and young adults. We addressed two key challenges in comparing TM and CE CPET among children, adolescents, and young adults: (1) the difficulty in quantifying the work performed in TM CPET, and (2) useful approaches to scaling CPET results when body size and physiologic function change so dramatically over the course of growth and development (Cooper et al., 1987; Cooper et al., 2014). CPET biomarkers are used to assess disease severity, progress, and response to therapy (including exercise prescriptions) across an expanding range of childhood diseases and conditions and across the lifespan (Ploeger et al., 2009; Pahkala et al., 2013; Liem et al., 2015; Sule and Fontaine, 2016; Cordingley et al., 2016; Gualano et al., 2017; Li et al., 2017). Despite these factors, CPET has failed to fulfil its promise in child health research and clinical practice (Ashish et al., 2015). A major barrier to more accurate and effective clinical use of CPET in children and adults has been a lack of harmonization of protocol types and exercise modalities (Ashish et al., 2015), factors that influence CPET results (Fredriksen et al., 1998; Beltrami et al., 2012; Bires et al., 2013; May et al., 2014; Cunha et al., 2015). For example, in clinical trials involving CPET in children over the past five years, a PubMed search revealed 40 published studies that used CE and 113 that used TM.

**Figure 1:**
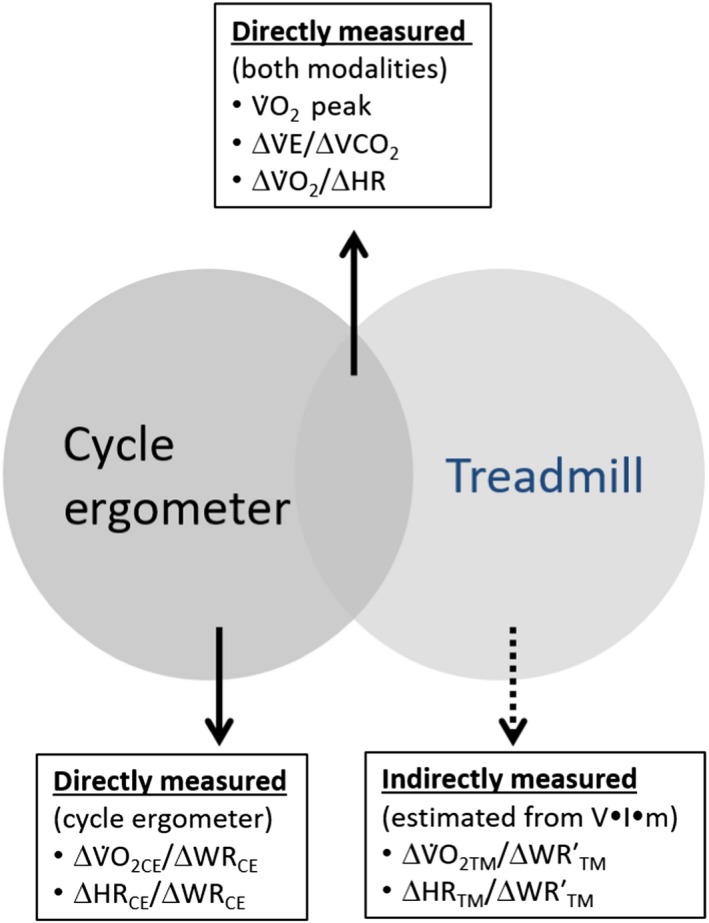
Overview – Interoperability challenge in CE and TM CPET. Some values are comparable (as Δ$\dot{V}$E/Δ$\dot{V}$CO_2_ and Δ$\dot{V}$O_2_/ΔHR) while others depend on accurate measurement of work rate (Δ$\dot{V}$O_2_/ΔWR and ΔHR/ΔWR) available only for the CE.

We concentrated on dynamic submaximal physiologic output variables (Fig. 1) that are less effort‐dependent than the traditional $\dot{V}$O_2_max and, arguably, more acceptable in children and adolescents, particularly those with chronic diseases or conditions (Stein et al., 2003; Cooper et al., 2014). Dynamic relationships among CPET variables (such as HR, $\dot{V}$E, $\dot{V}$CO_2_, and $\dot{V}$O_2_) reveal novel insights into cardiorespiratory function in health and disease (Cooper et al., 1984; Troutman et al., 1998; Moser et al., 2000; Chen et al., 2014; Elbehairy et al., 2015; Hestnes et al., 2017), and can be obtained in both CE and TM modalities without necessarily measuring work rate. The effect of exercise modality on submaximal physiologic output variables has not been adequately studied in children and adolescents.

In CE, the work rate is usually measured directly from the known resistance on the ergometer’s flywheel. $\dot{V}$O_2_ is linearly related to work rate (Whipp et al., 1981) and, as a consequence, CE protocols can be easily designed to produce a linear relationship between protocol duration (i.e., exercise time) and $\dot{V}$O_2_, which simplifies the ultimate analysis of CPET data. In contrast, it is difficult to estimate the relationship between work rate and $\dot{V}$O_2_ in TM because of the complexity of both the physics and human mechanical efficiency of treadmill walking and running (Workman and Armstrong, 1963; Kyröläinen et al., 1995; Porszasz et al., 2003; Keir et al., 2012; Azuma,^2014^). Treadmill work is determined by kinetic energy [functions of the velocity (V) of the TM and the body mass (M) of the participant] and work against gravity imposed by the TM grade or incline (Ruckstuhl et al., 2010). While body weight is often used to scale CPET values in an effort to compare results among individuals of different size, recent data suggest that LBM is significantly better correlated to size‐dependent CPET biomarkers (Cooper et al., 2014). Lean body mass (LBM) is a more direct measure of skeletal muscle, the predominant metabolizing tissue in exercise, than body weight. Consequently, we measured LBM using dual X‐ray absorptiometry DXA (Bridge et al., 2011), and used LBM in our comparison of the two CPET modalities.

Although the magnitude of peak or maximal $\dot{V}$O_2_ is similar in CE and TM CPET, one observation made consistently in both children and adults is that peak $\dot{V}$O_2_ tends to be somewhat greater in TM CPET (Turley and Wilmore, 1997). We hypothesized that CPET slope variables would reflect exercise modality differences as well. We further hypothesized that the metabolic response to TM is proportional to the kinetic energy exerted on the center of gravity of the body, therefore an estimate of TM work rate was calculated from body mass, V^2^, and TM incline would be linearly related to $\dot{V}$O_2_.

## Methods

### Participants

The study was approved by the UC Irvine Institutional Review Board. Inclusion criteria included healthy 7–35 y.o. participants (53% female) without any known respiratory, cardiac, or metabolic disease, and not taking any chronic prescribed medication. BMI of each participant was less than the 95th percentile for children (http://www.cdc.gov/healthyweight/assessing/bmi/chi) and BMI less than 30 for adults. Each volunteer visited the laboratory on two occasions. During the first visit, informed consent was obtained (parental consent + child assent for participants <18 y/o), demographic and anthropometric data were recorded, Tanner stage (by questionnaire) was assessed, and DXA was performed. A maximal progressive exercise protocol on a CE was also done on the first visit. At least 48 h later, each participant performed TM CPET with a protocol designed to mimic the rate of power increase found in the CE CPET.

### Anthropometric measurement and body composition

Standard calibrated scales and stadiometers were used to determine weight and height. Body composition, including LBM, fat mass, and percent body fat were determined by DXA using a Hologic QDR 4500 densitometer. Participants were scanned while lying supine and wearing light clothing. On the day of each test, the DXA instrument was calibrated using the procedures provided by the manufacturer, and DXA scans were performed and analyzed using pediatric software where appropriate.

### CE and TM protocols

The CE protocol consisted of a ramp‐type progressive cycle ergometry used previously in this and other laboratories to measure peak $\dot{V}$O_2_ in children and adults (Cooper et al., 2016). After a 2‐min period of unloaded cycling (0 W), power output was increased by 8–30 W/min. The increase in the ramp W/min was individualized for each participant empirically from the following formula:

(1) $$\text{work rate increment}=\left(\text{bodyweight}*3\right)/10$$

where work rate increment is in watts per minute and body weight in kg. This formula was derived empirically from the thousands of tests we have performed and is fairly reliable in producing CPET duration of 8–15 min, an interval previously determined to optimize evaluation of breath‐by‐breath data (Buchfuhrer et al., 1983; Myers and Froelicher, 1993). Participants cycled at a constant pedaling rate between 60 and 70 revolutions per minute (rpm) throughout the test on an electronically braked, servo‐controlled cycle ergometer. The increasing work rate was discontinued when the participants indicated that they had reached the limit of their tolerance and/or a drop occurred in pedaling rate below 60 rpm despite strong verbal encouragement. At this point, the work rate was lowered to 0 W and the participants continued to pedal for at least 5 more minutes while lowering the pedaling rate to below 40 rpm in order to prevent an excessively sudden drop in blood pressure (Kenney and Seals, 1993).

Since our goal was to compare the two modalities using tests in which $\dot{V}$O_2_ increased linearly over a duration of about 8–15 min, we used the results of the CE CPET (in which work rate was precisely known) to guide the velocity and incline configuration for the TM CPET performed on a separate data. Our coauthor (Dr. Porszasz) and coworkers (Porszasz et al., 2003) previously developed a TM CPET protocol that linearized the $\dot{V}$O_2_ increase over a 10–15 min exercise duration. They found that a protocol combining an initial slow walking speed that progressively increased in concert with a dynamically changing incline met the demands of an initially low exercise metabolic rate and optimum test duration.

In designing the protocol, we first determined the desired work rate for each of the 1‐min steps assuming a linear increase both in work rate and speed. The speed‐range we used was between 0.5 and 10.5 miles per hour (0.8 km/h and 16.8 km/h, respectively); baseline speed was set to 0.5 mph and every minute was increased by 0.5 mph up to 10.5 mph max. Each step had the same work rate and speed increments; having formulated these, the inclination was determined by the following formula:

(2) $$\text{TM inclination angle}=\arctan\left[\text{work rate}/\left(\text{mass}\times g\times v^{2}\right)\right]$$

where inclination angle is in radians; work rate in watts, mass in kg, g as 9.81m/sec^2^, and v in m/sec. The inclination as percentage was then determined and set for each step in the protocol. This resulted in a decreasing incline profile (set individualy, with 30% as the highest incline in the cohort), which approached 1% toward the end of the test. The general formula [Equation (2)] was used to calculate the desired change in TM incline to produce a given work rate at a particular velocity and work rate (Porszasz et al., 2003). We assumed that the work rate in TM CPET would be related to the kinetic energy equation, work = ½ mv^2^ and the following equation was used to estimate TM work rate:

(3) $$\text{WR}\left(\text{TM}\right)=k\cdot \text{mass}\cdot v^{2}\cdot \left(I+1\right)$$

where mass is body weight (kg), *v* is treadmill speed (m/sec), *I* is incline (%), and *k* is a conversion factor constant. We arbitrarily used the expression *I* + 1 because, at 0% incline, work rate would not be possible to calculate due to multiplication by zero. For convenience, we refer to *k*•mass•*v* ^2^• (*I* + 1) as VIM. Using these equations and protocol, we achieved a largely linear relationship between $\dot{V}$O_2_ and exercise work intensity up to a reasonable level and the test duration was between 8 and 15 min.

### Gas exchange measurement

Gas exchange was measured breath‐by‐breath using the SensorMedics metabolic system (Vmax Encore 229, Yorba Linda, CA). The breath‐by‐breath gas exchange data were interpolated to 1‐sec and 10‐sec bin averages were formed and used for all later analyses. Physiologically abnormal data for HR and gas exchange (e.g., HR < 50 beat/min or >230 beat/min, or $\dot{V}$O_2_ = 0 L/min or >5 L/min) and outliers, based on each subject, are occasionally observed in breath‐by‐breath CPET data obtained in children. These data were identified and excluded for slope or peak calculation.

### Calculation of submaximal CPET slopes and peak values

Submaximal slopes (Δ$\dot{V}$O_2_/ΔHR, Δ$\dot{V}$E/Δ$\dot{V}$CO_2_, etc.) were calculated using standard linear regression as described previously (Cooper et al., 2014) omitting the first minute and the last 30 sec of the exercise. The peak values were taken as the highest values in 20‐sec bin averages over the last 2 min of exercise. There is currently no validated, universally accepted approach for the determination of peak $\dot{V}$O_2_ in children. We used a criterion implemented in a large study by Rowland et al. (2008) defined by inability to maintain the pedaling cadence in association with subjective evidence of fatigue (sweating, hyperpnea) and HR >185 bpm (children) or >170 bpm (adults) and/or respiratory exchange ratio (RER, $\dot{V}$CO_2_/$\dot{V}$O_2_) >1.00 (children) or >1.10 (adults).

### Comparing CE and TM work rate input

We assumed that the linear relationship between work rate and $\dot{V}$O_2_ (Whipp et al., 1981) in both exercise modalities have the same slope and intercept, we used the linear regression parameters for the measured $\dot{V}$O_2_ on the CE (in which work rate was known) to determine the work rate on the TM (in which the velocity, incline, and participant’s weight were known) for each participant. The equivalent work rate on TM exercise was marked as WR’. The above is described adequately by the following two equations:

$$\begin{array}{cc} & \dot{V}{\text{O}}_{2}\left(\text{CE}\right)=a\cdot \text{WR}\left(\text{CE}\right)+\text{b}\\ & \;\;\;\text{and}\\ & \text{WR}\prime =\left[\dot{V}{\text{O}}_{2}\left(\text{TM}\right)-\text{b}\right]/\text{a} \end{array}$$

We used standard linear regression techniques to estimate the parameters ‘a’ and ‘b’ for each participant and calculated the WR’ for TM CPET.

### Comparison of fitness variables obtained from CE CPET and TM CPET

We compared the CPET variables described above obtained from the two modalities. To eliminate any confounding effect introduced by the estimation of WR’ (due to its dependence on the $\dot{V}$O_2_‐WR relationship derived from CPET‐CE), we also compared CPET values using VIM itself. In addition, we tested the degree to which CE and TM CPET variables scaled to body mass and composition, factors essential for understanding CPET in the growing child. For example, while the numerical value of ΔWR/ΔHR and ΔVIM/ΔHR will be quite different, we expected that their relationship and correlation to key variables such as age, body weight, lean body mass (LBM), and sex would be quite similar when comparing the two modalities.

### Statistical analysis

For each peak $\dot{V}$O_2_, ∆$\dot{V}$O_2_/∆HR slope, ∆$\dot{V}$E/∆$\dot{V}$CO_2_ slope, and other slopes, statistical comparisons of CE versus TM were performed using mixed models (via SAS PROC MIXED) to account for subject level intercorrelation between the paired modality measurements (TM and CE). Each model also included puberty group (children tanner 1–2, adolescents 4‐5, adults >18 years), sex, puberty × sex, puberty group × modality interaction, sex × modality interaction, and puberty group × sex × modality interaction. Post hoc comparisons of model‐generated, least‐square (LS) means were evaluated where significant fixed effects were found. This was done according to the hierarchy principle such that if an interaction was present, only the appropriate conditional means were compared and interpreted. Significance for the post hoc comparisons was determined by Tukey‐adjusted *P*‐values of the LS mean differences. We performed a standard Bland‐Altman (BA) analysis to compare to peak VO_2_ and submaximal slopes between the two modalities.

## Results

### Participants characteristics

**Figure 2:**
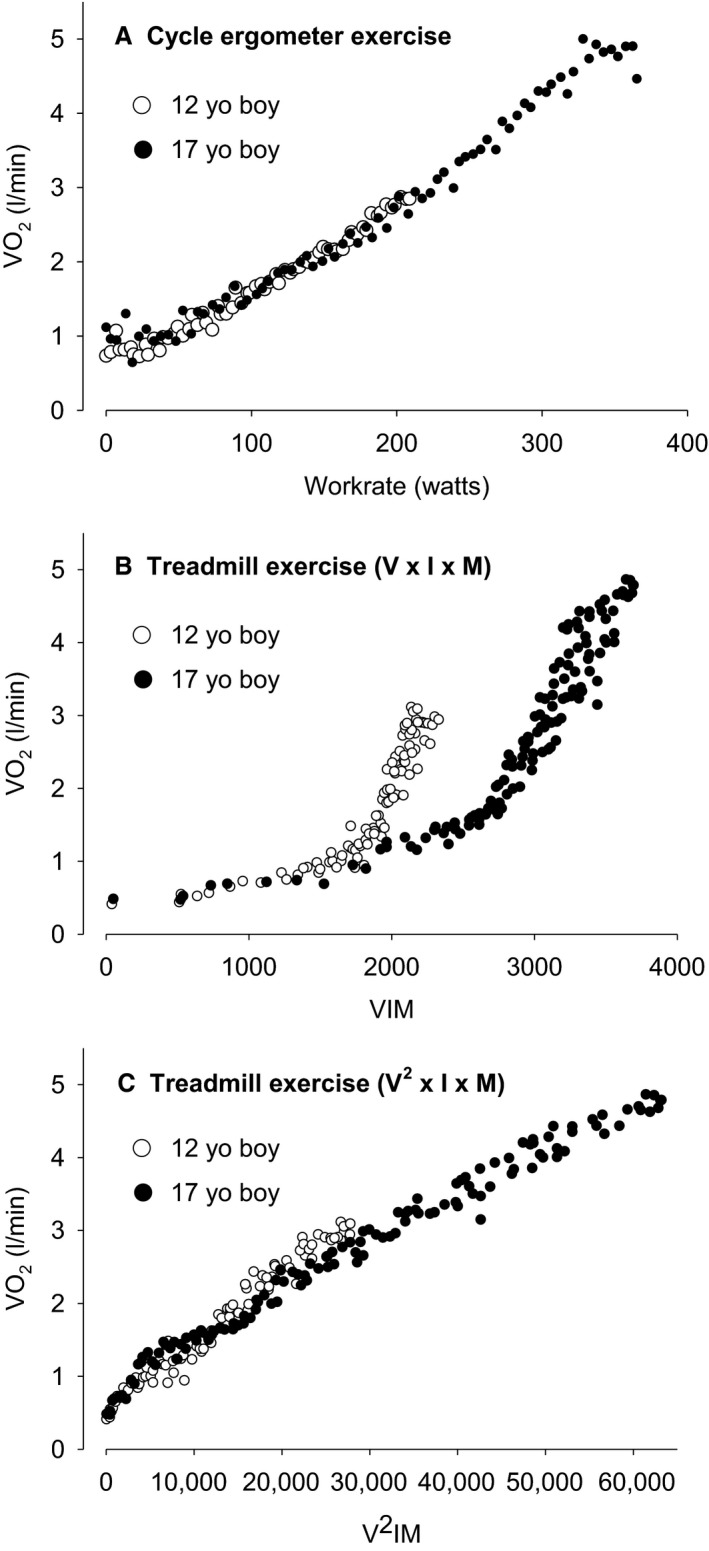
(A) Relationship between $\dot{V}$O_2_ and work rate in early (12 y/o) boy and late (17 y/o) pubertal boy in CE CPET. (B) Relationship between $\dot{V}$O_2_ and *V* × *I* × *M* (see text) in TM CPET in the two participants. (C) Relationship between $\dot{V}$O_2_ and *V* ^2^ × I × M (see text) in TM CPET in the two participants. By using this approach, we were able to linearize the relationship between oxygen uptake and velocity, incline and mass.

Representative examples of CE and TM CPET are shown in Figure 2. A total of 111 healthy children and young adults (7–34 y.o.) participated in this study. We excluded 17 of them from the final analysis: five due to technical problems, three due to incompletion of the study protocol, two due to a submaximal effort on the TM, six due to inability to assess the Tanner score, and one due to exercise‐induced bronchoconstriction following CE ramp test. Ninety‐four participants were included in the analysis, and demographic and anthropometric data are presented in Table 1. For analysis of peak $\dot{V}$O_2_, data from 88 participants were analyzed (six participants did not meet the criteria for a maximal test as noted above). Submaximal slopes, and peak CPET values are shown in Tables 2, 3, 4. Detailed summary of statistical analyses are shown in Tables 5 and 6.

**Table 1:** Participant characteristics.

Group	Children	Children	Adolescent	Adolescent	Adult	Adult
Sex	M	F	M	F	M	F
Tanner	1–2	1–2	4–5	4–5	NA	NA
*N*	18	16	18	23	8	11
Age (year)	10.8 ± 1.7	8.8 ± 1.1	16.9 ± 1.4	15.5 ± 1.8	29.0 ± 2.3	26.3 ± 4.6
Height (cm)	144.0 ± 11.8	132.1 ± 9.4	172.4 ± 7.0	161.4 ± 6.0	178.8 ± 9.0	161.8 ± 4.8
Total body mass (kg)	37.4 ± 12.0	29.7 ± 8.0	62.4 ± 9.4	54.8 ± 8.4	79.5 ± 8.3	58.1 ± 7.6
Lean body mass (kg)	25.2 ± 6.5	19.0 ± 3.8	47.3 ± 6.5	35.4 ± 4.6	59.5 ± 7.0	38.4 ± 5.0
% Body fat	29.0 ± 5.9	32.6 ± 5.6	21.3 ± 5.0	32.6 ± 5.2	22.5 ± 5.2	31.2 ± 4.3
BMI (kg/m^2^)	17.5 ± 3.0	16.7 ± 2.4	17.2 ± 3.3	20.9 ± 2.2	24.9 ± 1.9	22.1 ± 2.3
BMI percentile	47.3 ± 30.7	49.4 ± 29.0	49.9 ± 29.4	43.1 ± 23.5	N/A	N/A
Ethnicity (Hispanic or Latino)	0	1	1	2	0	0
Race (White/Asian/African‐American)	16/1/1	13/2/1	8/10/0	16/7/0	5/3/0	7/3/1

M, Male, F, Female, BMI, body mass index.

**Table 2:** Submaximal slopes of CPET variables obtainable from both CE and TM modalities.

Group	Children	Children	Adolescent	Adolescent	Adult	Adult
Sex	M	F	M	F	M	F
CE Δ$\dot{V}$E/Δ$\dot{V}$CO_2_	31.4 ± 3.5	31.2 ± 2.7	27.9 ± 4.0	30.7 ± 4.0	27.2 ± 3.9	29.4 ± 5.4
TM Δ$\dot{V}$E/Δ$\dot{V}$CO_2_	30.6 ± 2.2	29.8 ± 2.7	27.9 ± 2.2	28.7 ± 2.5	25.2 ± 3.7	28.9 ± 3.7
CE Δ$\dot{V}$O_2_/ΔHR (mL/beat)	13.7 ± 4.1	9.4 ± 1.9	26.0 ± 6.4	15.8 ± 4.8	29.2 ± 8.0	21.2 ± 5.9
TM Δ$\dot{V}$O_2_/ΔHR (mL/beat)	14.9 ± 4.6	10.8 ± 2.6	27.2 ± 7.1	18.8 ± 4.27	31.7 ± 7.6	22.0 ± 5.0

**Table 3:** Similar maturation and sex patterns in heartrate response to work rate in CE CPET (work rate actually measured) and TM CPET (work rate estimated by WR′ and VIM).

Group	Children	Children	Adolescent	Adolescent	Adult	Adult
Sex	M	F	M	F	M	F
CE ΔWR/ΔHR (watts/beat/min)	1.17 ± 0.41	0.77 ± 0.19	2.33 ± 0.49	1.58 ± 0.34	2.61 ± 0.62	2.09 ± 0.61
TM ΔWR′/ΔHR (watts/beat/min)	1.29 ± 0.48	0.90 ± 0.27	2.46 ± 0.55	1.92 ± 0.43	2.85 ± 0.61	2.20 ± 0.64
TM ΔVIM/ΔHR	123.9 ± 43.4	85.9 ± 22.7	334.2 ± 130.2	198.1 ± 75.5	368.9 ± 110.6	245.1 ± 78.9

Data are presented as mean ± SD.

For CE WR‐HR and TM WR′‐HR, the sex difference is significant for all three puberty groups. For VIM‐HR the sex difference is significant for adolescents and adults, but not children. In males, all three measures, demonstrate a puberty effect with adolescents and adults having larger slopes than children. Adolescents and adult males are not significantly different from each other. In females all three measures also show adolescents and adults having larger slopes than children. In addition, CE WR‐HR and TM VIM‐HR show adult females having significantly larger slopes than adolescent females, but this was not the case for TM WR′‐HR.

**Table 4:** Peak $\dot{V}$O_2_ and HR from CE and TM modalities.

Group	Children	Children	Adolescent	Adolescent	Adult	Adult
Sex	M	F	M	F	M	F
CE peak $\dot{V}$O_2_ (L/min)	1.89 ± 0.47	1.31 ± 0.27	3.45 ± 0.69	2.20 ± 0.53	3.81 ± 0.87	2.52 ± 0.63
CE peak $\dot{V}$O_2_/weight (mL/kg/min)	51.5 ± 7.2	45.0 ± 7.3	54.3 ± 6.8	40.5 ± 8.0	47.8 ± 9.0	43.4 ± 10.0
CE peak $\dot{V}$O_2_/LBM (mL/kg/min)	74.9 ± 6.1	70.1 ± 6.7	71.0 ± 7.2	62.2 ± 10.6	63.7 ± 10.4	62.2 ± 11.9
TM peak $\dot{V}$O_2_ (L/min)‐	1.93 ± 0.56	1.37 ± 0.27	3.42 ± 0.48	2.41 ± 0.49	4.29 ± 0.64	2.64 ± 0.43
TM peak $\dot{V}$O_2_/weight (mL/kg/min)	52.2 ± 8.1	47.3 ± 7.7	55.5 ± 7.3	44.3 ± 7.6	54.0 ± 5.7	45.9 ± 8.2
TM peak $\dot{V}$O_2_/LBM (mL/kg/min)	76.1 ± 8.9	73.2 ± 9.1	72.6 ± 7.5	68.0 ± 8.9	72.1 ± 5.8	69.0 ± 9.2
CE peak HR (beats/min)	199.8 ± 7.7	194.5 ± 9.9	197.6 ± 11.1	193.3 ± 8.5	189.3 ± 10.9	183.9 ± 8.8
TM peak HR (beats/min)	195.8 ± 7.0	193.3 ± 9.8	198.8 ± 8.6	197.3 ± 7.5	193.3 ± 12.1	187.5 ± 9.2
CE protocol duration	669.4 ± 72.7	577.9 ± 118.1	691.6 ± 97.2	601.6 ± 70.0	689.8 ± 66.8	604.9 ± 62.2
TM protocol duration	700.3 ± 105.1	581.2 ± 92.1	951.0 ± 153.7	785.8 ± 135.4	1013.1 ± 140.8	805.9 ± 169.7

**Table 5:** Fixed effects from mixed model analysis.

CPET variable	Δ$\dot{V}$O_2_/ΔHR		Δ$\dot{V}$E/Δ$\dot{V}$CO_2_		Peak $\dot{V}$O_2_/weight		Peak $\dot{V}$O_2_/LBM	
Effect	*F*‐stat	*P*	*F*‐stat	*P*	*F*‐stat	*P*	*F*‐stat	*P*
Puberty	107.15	<0.0001	12.03	<0.0001	0.28	0.7549	5.13	0.0078
Sex	85.50	<0.0001	8.16	0.0053	44.09	<0.0001	9.88	0.0023
Modality	4.34	0.0402	4.77	0.0317	5.10	0.0266	7.04	0.0095
Puberty × sex	4.89	0.0098	4.15	0.019	3.90	0.024	2.26	0.1108
Puberty × modality	0.09	0.9098	0.02	0.9816	0.37	0.6907	0.71	0.4968
Sex × modality	0.00	0.9456	0.09	0.7664	0.00	0.9543	0.22	0.6406
Puberty × Sex × Modality	0.33	0.7227	0.78	0.4597	0.53	0.5881	0.88	0.4177

**Table 6:** Paired comparisons of mixed model least square means.

CPET variable	Δ$\dot{V}$O_2_/ΔHR (mL/beat)		Δ$\dot{V}$E/Δ$\dot{V}$CO_2_		Peak $\dot{V}$O_2_ per weight (mL/min/kg)		Peak $\dot{V}$O_2_ per LBM (mL/min/kg)	
	Mean diff	*P*	Mean diff	*P*	Mean diff	*P*	Mean diff	*P*
Pubertal status (child = C; adolescent = ADO; adult = ADU								
C vs. ADO	−9.725	<0.0001	2.110	0.0003	0.360	0.7875	3.674	0.0149
C vs. ADU	−13.824	<0.0001	3.105	<0.0001	1.201	0.457	5.334	0.0046
ADO vs. ADU	−4.099	0.0002	0.995	0.1436	0.841	0.5906	1.659	0.3545
Sex (M,F)								
M vs. F	7.448	<0.0001	−1.506	0.0053	8.153	<0.0001	4.380	0.0023
Modality								
CE vs. TM	−1.679	0.0402	1.151	0.0317	−2.773	0.0266	−3.697	0.0095
Pubertal status × sex								
C: M vs. F	4.219	0.0009	0.539	0.5153	5.673	0.005	*	*
ADO: M vs. F	9.263	<0.0001	−2.130	0.0060	12.523	<0.0001	*	*
ADU: M vs. F	8.861	<0.0001	−2.926	0.0103	6.263	0.0159	*	*
M: C vs ADO	−12.247	<0.0001	3.444	<0.0001	−3.064	0.1231	*	*
M: C vs. ADU	−16.144	<0.0001	4.837	<0.0001	0.907	0.7061	*	*
M: ADO vs. ADU	−3.898	0.0149	1.393	0.1759	3.971	0.0976	*	*
F: C vs ADO	−7.203	<0.0001	0.776	0.3240	3.785	0.0384	*	*
F: C vs. ADU	−11.503	<0.0001	1.372	0.1483	1.496	0.4871	*	*
F: ADO vs. ADU	−4.299	0.0023	0.597	0.5000	−2.289	0.2607	*	*

* No significant interactions.

### Linearity of $\dot{V}$O_2_ with work rate estimate and exercise duration in the two modalities

Corroborating the previous work by Porszasz et al., (2003, we achieved success in linearizing the relationship between $\dot{V}$O_2_ and exercise duration (time). This was evidenced both by visual inspection of the exercise tests (e.g., Fig. 2) as well as by the remarkably high correlation between $\dot{V}$O_2_ and time for the TM CPET (mean *r* was 0.978). We compared the correlation coefficients of two linear regressions: $\dot{V}$O_2_ versus *v* × (I + 1) × mass and *v* ^2^ × (*I* + 1) × mass. For the former, the average *R*_l_^2^ = 0.7889 and for the latter *R_s_* ^2^ = 0.9255. A paired *t*‐test for the mean difference was statistically significant [Δ(*R* ^2^ *_s_*‐*R* ^2^ *_l_*) = 0.1367, *P* < 0.00001], suggesting stronger prediction of $\dot{V}$O_2_ by *V* ^2^ *IM*. The duration of exercise for TM and CE modality in each group of participants is shown in Table 4. For the adolescents and young adults, TM duration was significantly longer than CE. In general, exercise duration for both modalities was longer in males then in females.

### Submaximal CPET variables

#### ∆$\dot{V}$O_2_/∆HR

**Figure 3:**
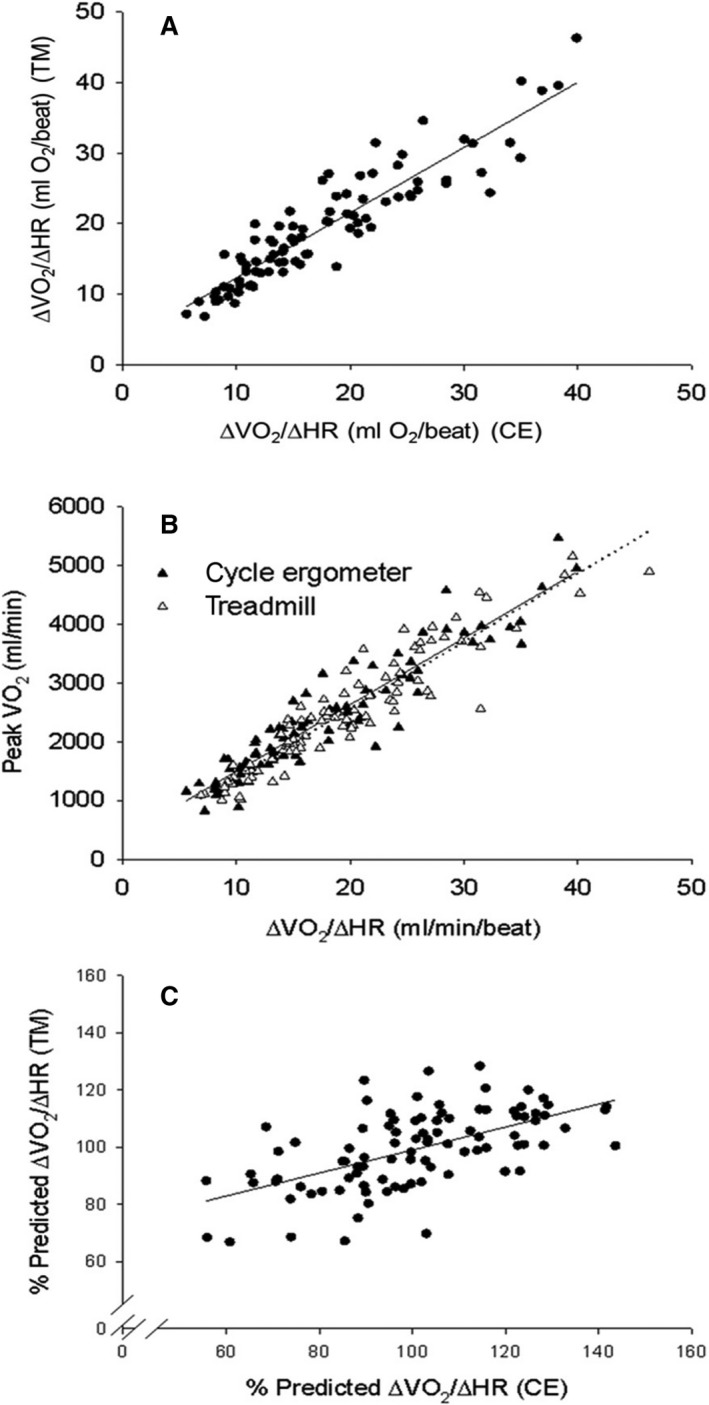
Interoperability of Δ$\dot{V}$O_2_/ΔHR derived from CPET‐CE and CPET –TM. (A) The slope of the linear regression equation was highly significant, 0.923 ± 0.0419, *P* < 0.0001; the *y*‐intercept, 3.11 ± 0.82 mL O_2_/beat, was significant at *P* = 0.0003, and *r* = 0.92. (B) Relationship of CE and TM Δ$\dot{V}$O_2_/ΔHR to peak $\dot{V}$O_2_. Both modalities revealed very high correlations. Linear regression parameters for CPET CE (solid line) were: peak $\dot{V}$O_2_ (mL/min) = 113.3 × Δ$\dot{V}$O_2_/ΔHR (mL/beat) + 361.7, *r* = 0.94; and for CPET TM (dotted line) peak $\dot{V}$O_2_ (mL/min) = 115.2 × Δ$\dot{V}$O_2_/ΔHR (mL/beat) + 247.0, *r* = 0.94. (C) We calculated the percent predicted peak $\dot{V}$O_2_ based on the LBM linear regression (see text) and plotted TM percent predicted vs. CE percent predicted peak $\dot{V}$O_2_. The correlation coefficient was *r* = 0.0.66, *P* < 0.0001.

∆$\dot{V}$ O_2_/∆HR results are shown in Figure 3 and Table 2. The CE and TM values were highly correlated (*P* < 0.0001, Fig. 3A). A small but significantly higher mean ∆$\dot{V}$O_2_/∆HR difference (1.7 ± 0.81 mL/beat, about 10%) was found in TM versus CE. For the group as a whole, BA analysis revealed higher ∆$\dot{V}$O_2_/∆HR for TM CPET (bias of 1.74, 95% CI of 1.06 to 2.42). Statistically significant maturation‐dependent differences were observed in both males and females (Table 6). Consistent with our previous study of cycle ergometer exercise in children and adolescents (Cooper et al., 2014), ∆$\dot{V}$O_2_/∆HR increased with LBM (*r* = 0.88, *P* < 0.0001). For both CE and TM (Fig. 3B), peak $\dot{V}$O_2_ was highly correlated to ∆$\dot{V}$O_2_/∆HR. We used the linear regression equations relating ∆$\dot{V}$O_2_/∆HR and LBM to calculate a predicted value for each participant, then compared the percent predicted from the CE and TM CPET to determine how interchangeable the two modalities were. A moderate correlation was found (correlation coefficient *r* = 0.66, *P* < 0.0001, Fig. 3C).

#### ∆$\dot{V}$E/∆$\dot{V}$CO_2_

**Figure 4:**
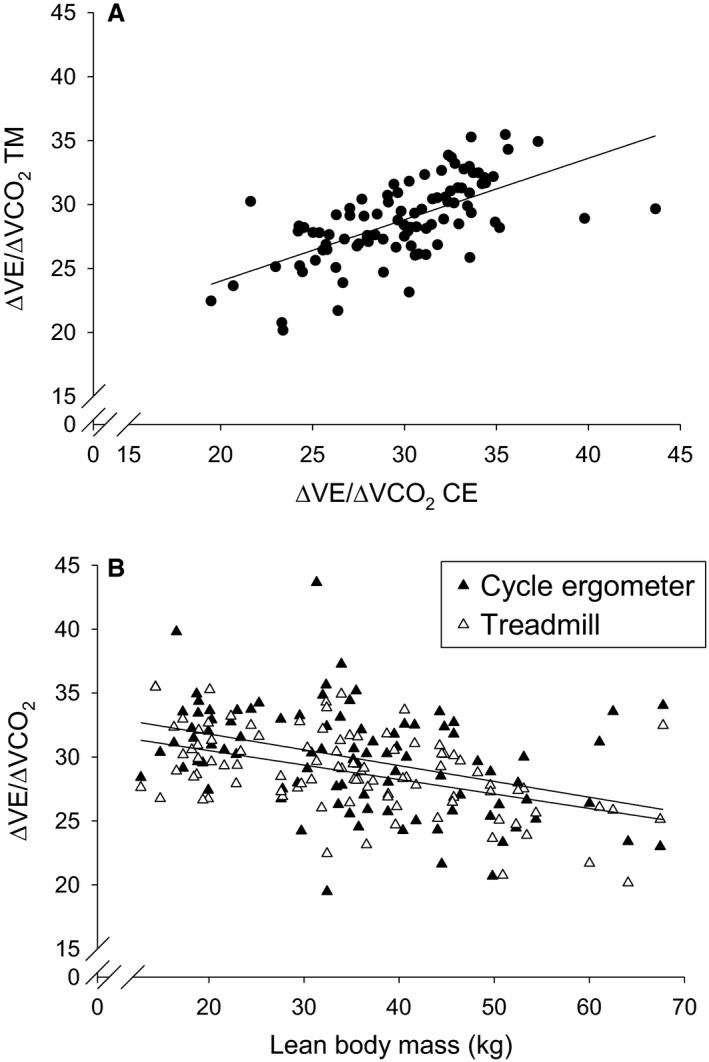
Interoperability of Δ$\dot{V}$E/Δ$\dot{V}$CO_2_ derived from CPET‐CE and CPET –TM. (A) The slopes were significantly correlated Δ$\dot{V}$E/Δ$\dot{V}$CO_2_ (TM) = 0.48 × Δ$\dot{V}$E/Δ$\dot{V}$ CO_2_ (CE) + 14.4, *r* = 0.63, ‐* P* < 0.0001. (B) The slopes from both modalities were inversely correlated with LBM (CE: Δ$\dot{V}$E/Δ$\dot{V}$ CO_2_ = –0.12 × LBM (kg) + 34.3, *r* = −0.40, *P* < 0.0001; TM: Δ$\dot{V}$E/Δ$\dot{V}$CO_2_ = –0.11 × LBM (kg) + 32.3, *r* = −0.48, *P* < 0.0001. These data corroborate previous studies showing generally higher Δ$\dot{V}$E/Δ$\dot{V}$ CO_2_ in younger (smaller) children compared with adolescents and young adults.

∆$\dot{V}$E/∆$\dot{V}$CO_2_ results are shown in Figure 4 and Table 2. The CE and TM values were significantly correlated (*P* < 0.0001, Fig. 4A). A small but significantly higher mean ∆$\dot{V}$E/∆$\dot{V}$CO_2_ difference (1.151 ± 0.527, about 3.7%) was found in CE vs. TM. For the group as a whole, BA analysis revealed lower ∆$\dot{V}$E/∆$\dot{V}$CO_2_ in TM CPET (bias of –1.13, 95%CI of –1.79 to –0.47). In the males only, the values were significantly greater (*P* < 0.0001) in the children compared to the adolescents and the adults. For the participant population as a whole, ∆$\dot{V}$E/∆$\dot{V}$CO_2_ was inversely correlated with LBM (*P* < 0.0001, Fig. 4B).

### *Sex effects on ∆* $\dot{V}$ *O_2_/∆HR and ∆* $\dot{V}$ *E/∆* $\dot{V}$ *CO_2_*

Both CE and TM CPET revealed significant sex effects. *∆* $\dot{V}$ *O_2_/∆HR* was greater in males, and *∆* $\dot{V}$ *E/∆* $\dot{V}$ *CO_2_* was greater in females. The sex effect was not influenced by CPET modality.

### Relationships between HR, WR, WR′, and VIM

**Figure 5:**
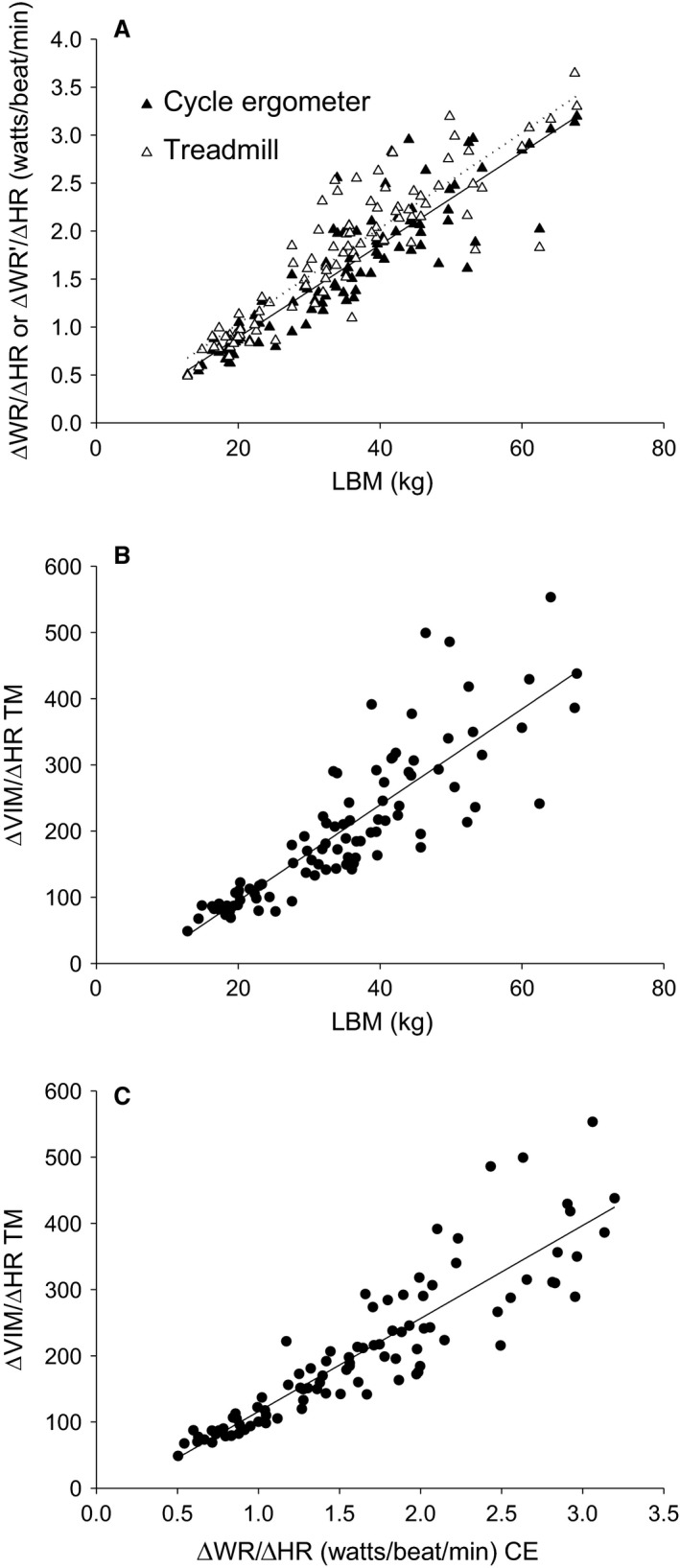
Interoperability of work rate–heart rate slopes derived from CPET‐CE and CPET‐TM. (A) The slopes for ΔWR/ΔHR and ΔWR′/ΔHR as a function of LBM were similar: ΔWR/ΔHR (CE) = 0.048 × LBM–0.070, *r* = 0.89, *P* < 0.0001; ΔWR′/ΔHR (TM) = 0.050 × LBM + 0.041, *r* = 0.89, *P* < 0.0001. (B) Using data derived solely from CPET TM showed a very similar relationship, ΔVIM/ΔHR (TM) = 7.24 × LBM–50.12, *r* = 0.86, *P* < 0.0001. (C) Dynamic WR‐HR data obtained from the two modalities were highly correlated. ΔVIM/ΔHR (TM) = 140.4 × ΔWR/ΔHR (CE)–24.5, *r* = 0.90, *P* < 0.0001.

As shown in Figure 5, we found strong correlations between LBM and either ∆WR/∆HR from CE CPET or ∆WR’/∆HR from TM CPET (Fig. 5A). ∆VIM/∆HR from TM CPET was highly correlated to LBM and to ∆WR/∆HR from CE CPET (Fig. 5B and 5, respectively). As shown in Table 3, the WR, WR’, and VIM relationships with HR all reflected comparable patterns within the subpopulations of the participants.

### Peak $\dot{V}$O_2_ comparison: CE vs. TM and relationship to submaximal CPET variables

**Figure 6:**
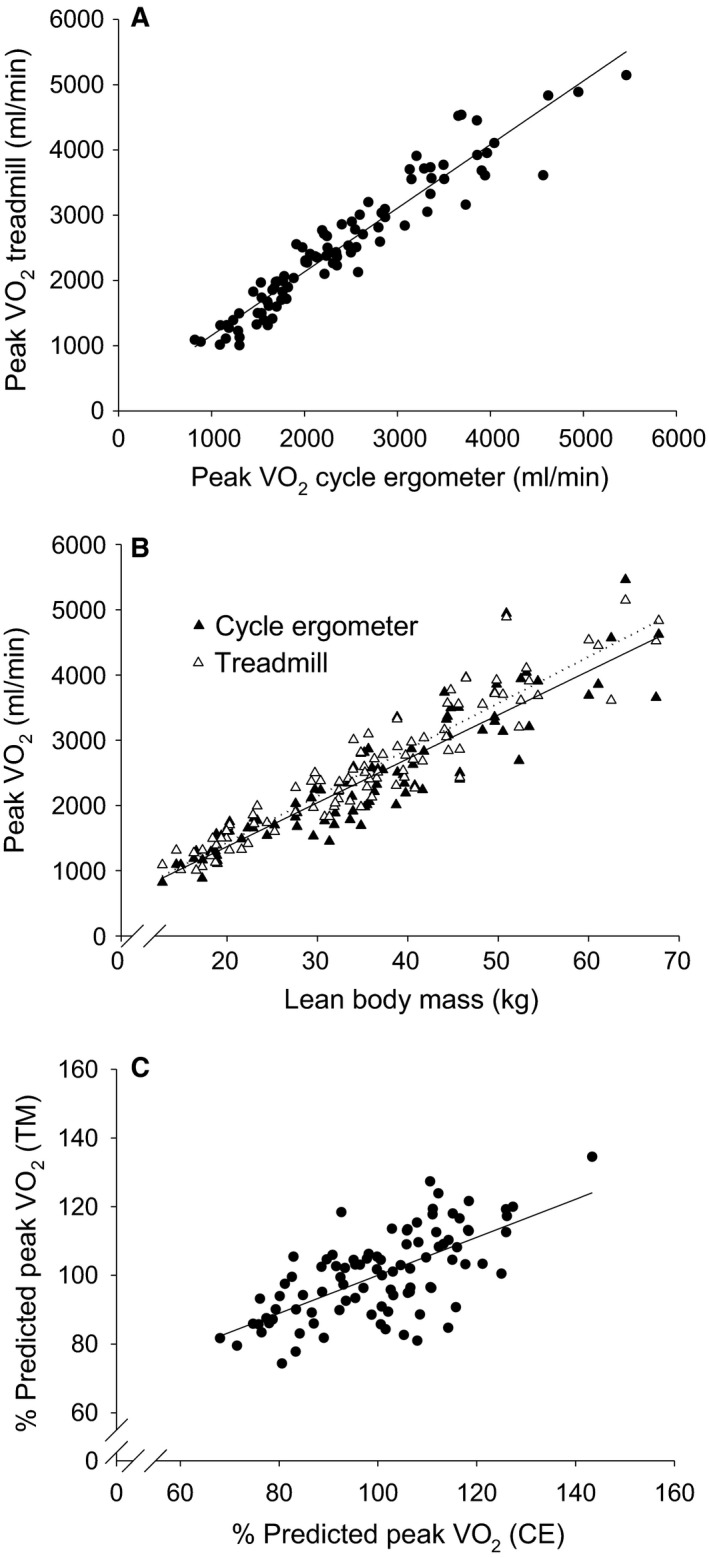
Interoperability of peak $\dot{V}$O_2_ derived from CPET‐CE and CPET –TM. (A) The slope of the linear regression equation was highly significant, 0.975 ± 0.0316, *P* < 0.0001; the y‐intercept, 182.81 ± 81.87 mL O_2_/min, was significant at *P* = 0.028, and *r* = 0.95. (B) Relationship of CE and TM peak $\dot{V}$O_2_ to lean body mass. Both modalities revealed very high correlations. Linear regression parameters for CPET CE (solid line) were: peak $\dot{V}$O_2_ CE (mL/min) = 67.3 × LBM (kg) + 21.8, *r* = 0.91; and for CPET TM (dotted line) peak $\dot{V}$O_2_ TM (mL/min) = 71.3 × LBM (kg) + 4.39, *r* = 0.94. The correlation coefficients for LBM were higher than for weight in both modalities: CE, peak $\dot{V}$O_2_ = 26.4 × Weight + 46.4, *r* = 0.82; TM, peak $\dot{V}$O_2_ = 49.6 × Weight–13.0, *r* = 0.86. (C) We calculated the percent predicted peak $\dot{V}$O_2_ based on the LBM linear regression and plotted TM percent predicted versus CE percent predicted peak $\dot{V}$O_2_. The correlation coefficient was *r* = 0.66.

Figure 6 and Table 4 shows the correlations between peak $\dot{V}$O_2_ for the whole participant population from the two modalities. Peak $\dot{V}$O_2_ was highly correlated between CE and TM (Fig. 6A), and both CE and TM CPET peak $\dot{V}$O_2_ demonstrated high correlation with LBM (correlations with weight were high, but not as high as with LBM, Fig. 6B). Overall, a small (5.9 ± 1.3%) but significantly higher mean peak $\dot{V}$O_2_ difference was found in TM. For the group as a whole, BA analysis revealed higher peak $\dot{V}$O_2_ for TM CPET (bias of 122 mL/min, 95% CI of 62–184 mL/min). However, within the puberty subgroups, there was no significant difference between CE and TM. Males had higher peak $\dot{V}$O_2_ than females at all puberty levels. In males, there were no differences among puberty groups. In females, adolescents had the lowest mean values, statistically significant only younger ages.

We used the linear regression equations relating peak $\dot{V}$O_2_ and LBM to calculate a predicted value for peak $\dot{V}$O_2_, then compared the percent predicted from the CE and TM CPET to determine how interchangeable the two modalities were. A moderate correlation was found (Fig. 6C).

## Discussion

For TM CPET, we were able to design a protocol that linearized the relationship of $\dot{V}$O_2_ to both exercise duration and an estimate of work rate using the participant’s body weight and data easily obtained from TM CPET, namely TM speed and incline. The dynamic relationship between the novel VIM estimate of work rate and CPET variables like HR and $\dot{V}$O_2_ paralleled the relationships we found using CE CPET in which work rate is measured directly. This can provide investigators with new tools to gauge fitness in children and adolescents using TM CPET. Dynamic submaximal CPET variables (such as Δ$\dot{V}$O_2_/ΔHR and ∆$\dot{V}$E/∆$\dot{V}$CO_2_) were highly correlated between the new linear TM and CE CPET protocols: this is the first attempt to analyze these submaximal CPET parameters in a cohort of children, adolescents, and young adults. Furthermore, we found that the relationship of these CPET results to critical exercise‐response determinants such as body size were similar in both exercise modalities. Although the HR and gas exchange results of TM and CE exercise were comparable, our data corroborated previous work establishing that CPET TM peak $\dot{V}$O_2_ is somewhat and significantly greater than CE CPET. We extended this finding to a submaximal CPET variable, Δ$\dot{V}$O_2_/ΔHR. The dynamic submaximal relationship between $\dot{V}$E and $\dot{V}$CO_2_, (∆$\dot{V}$E/∆$\dot{V}$CO_2_) was to a small but significant degree higher in CE CPET.

The mechanisms responsible for the larger Δ$\dot{V}$O_2_/ΔHR in TM CPET are not clearly evident. The Fick equation [$\dot{V}$O_2_ = HR•SV × (*a* − v̄)O_2,_ where HR is heart rate, SV is stroke volume, and (*a* − v̄)O_2_ is arteriovenous oxygen content difference] indicates that a greater increase in $\dot{V}$O_2_ per given change in HR can occur only as a result of a greater change in SV or widening of the arteriovenous O_2_ concentration difference. A possible mechanism influencing stroke volume could be higher venous return and increased muscle mass involved in exercise during TM versus CE. We reanalyzed the data cited earlier from Turley et al. (1997) who measured (*a* − v̄)O_2_ and SV indirectly and noninvasively in 24 children and 24 adults during both TM and CE progressive exercise. Interestingly, while we could find no systematic differences in SV between cycle and treadmill exercise, we did find that the average (*a* − v̄)O_2_ during exercise was significantly (*P* < 0.01) higher in TM exercise (10.7/100 mL) compared with CE exercise (9.6/100 mL). Further studies will be needed to examine the matching of blood flow distribution in the exercising muscle to determine possible mechanisms leading to greater O_2_ extraction during TM exercise, leading to the small but significant differences in the Δ$\dot{V}$O_2_/ΔHR.

Δ$\dot{V}$E/Δ$\dot{V}$CO_2_ values obtained from TM and CE CPET were correlated, but not as strongly as the Δ$\dot{V}$O_2_/ΔHR CPET variable (Fig. 3A, 4A). The relationship of $\dot{V}$E to $\dot{V}$CO_2_ during exercise reveals useful clinical information regarding respiratory dead space and the systemic set point of CO_2_ concentration that ultimately modulates respiratory control centers in the brainstem and carotid bodies (Armon et al., 1991; Rausch et al., 1991). Clinical insights using CPET‐derived Δ$\dot{V}$E/Δ$\dot{V}$CO_2_ have been gained in children and adults from both TM and CE CPET in diseases ranging from cystic fibrosis to heart failure (Moser et al., 2000; Ingle et al., 2012). We did observe small but statistically significant differences in Δ$\dot{V}$E/Δ$\dot{V}$CO_2_, for example, a 3.7% larger value overall for CE exercise. Our study was not configured to determine the mechanism of this difference (e.g., greater ventilatory dead space or a lower CO_2_ set point in CE compared with TM CPET). Nonetheless, while the differences were small in this cohort of children and adults with no history of lung disease, one might speculate that variables like Δ$\dot{V}$E/Δ$\dot{V}$CO_2_ might become more useful in participants with chronic lung disease.

One reason for the somewhat smaller correlation for the Δ$\dot{V}$E/Δ$\dot{V}$CO_2_ variable between the two modalities may be that the range of Δ$\dot{V}$E/Δ$\dot{V}$CO_2_ values in our cohort was substantially smaller than for other CPET variables. For example, Δ$\dot{V}$O_2_/ΔHR ranged from about 7–45 mL O_2_/beat while Δ$\dot{V}$E/Δ$\dot{V}$CO_2_ ranged only from about 25–35 (unitless). Within a participant group of healthy individuals with no history of lung or heart disease, a major determinant of many CPET variables is body size, particularly muscle mass (Cooper et al., 2014). Previous studies using CE CPET demonstrated high correlations between body size and Δ$\dot{V}$O_2_/ΔHR and weak, but significant, inverse correlations with Δ$\dot{V}$E/Δ$\dot{V}$CO_2_.

We found that the relationships between Δ$\dot{V}$O_2_/ΔHR and body mass in TM CPET paralleled the relationships we found previously using CE CPET. The correlation between TM CPET‐derived Δ$\dot{V}$O_2_/ΔHR and body weight was strong, but even stronger when correlated with LBM. These results emphasize the need to scale Δ$\dot{V}$O_2_/ΔHR to some metric of body size in order to interpret the results correctly. The TM CPET‐derived Δ$\dot{V}$E/Δ$\dot{V}$CO_2_ was to a small but significant degree inversely correlated to body size, similar to the earlier studies using CE CPET (Cooper et al., 1984; Nagano et al., 1998). These similar results from the two different exercise modalities bolster the idea that physiologic mechanisms, such as the CO_2_ set point or the relationship between dead space and tidal volume, systematically change over childhood and adolescence.

Both modalities revealed similar and significant sex effects in Δ$\dot{V}$O_2_/ΔHR and Δ$\dot{V}$E/Δ$\dot{V}$CO_2_. The higher oxygen extraction per beat found in male participants reflects, as noted above, the influence of stroke volume and (*a* − v̄)O_2_. In adults, left ventricular size is smaller in females compared to males (Gebhard et al., 2013). Similar observations have been made in children (Vinet et al., 2003). These results might explain the sexual dimorphism of the Δ$\dot{V}$O_2_/ΔHR. Although not as well studied as heart size, one study in young and middle‐aged adults also showed generally higher $\dot{V}$E‐$\dot{V}$CO_2_ based parameters in females compared with males (Sun et al., 2002), an observation not seen in one exercise study in younger volunteers (Guerrero et al., 2008). Sexual dimorphism in respiratory control in adults is a known phenomenon, but the impact of sex on respiratory control during exercise in children is not well understood. Whether the generally higher Δ$\dot{V}$E/Δ$\dot{V}$CO_2_ that we found in females indicate greater deadspace ventilation or, alternatively, a lower CO_2_ set point, has yet to be determined.

### CPET typically consists of an ergometer programmed to increase the participant’s

work rate coupled with a set of devices capable of measuring physiologic responses such as gas exchange or HR. These physiologic outputs are useful only insofar as they can be scaled. For example, an isolated HR measured during exercise is uninterpretable unless it is dynamically scaled to a CPET input such as the work rate. We used several approaches to address the challenging problem of estimating work performed during TM CPET. There are very compelling reasons to do this; one of the most potentially impactful would be in reanalyzing fitness data from many studies in children in which TM CPET in some form is used to estimate, rather than measure, peak $\dot{V}$O_2_ [e.g., NHANES (Astrand and Ryhming, 1954; Jackson et al., 1990)]. Subsequent calculated estimates of $\dot{V}$O_2_max derived from the submaximal CPET may include variables or constants reflecting levels of habitual physical activity or normative values obtained from studies in adults. Such approaches can contribute to the increasingly recognized problems that confound data interpretation due to misspecification, collinearity, and mathematical coupling (Tu et al., 2004; Aggarwal and Ranganathan, 2016).

An analysis of TM CPET that relies predominantly on actually measured data would advance our ability to accurately gauge fitness from CPET. In the current study, we were able to calculate the WR’ TM exercise based on the $\dot{V}$O_2_‐WR relationship measured during CE CPET. As shown in Figure 5A, ΔWR/ΔHR from CE CPET and ΔWR′/ΔHR from TM CPET had virtually identical relationships with LBM (and body weight, data not shown). Additional parallel effects of sex are shown in Table 4.

Using the VIM estimate of TM work rate led to a linear relationship with $\dot{V}$O_2_ (Fig. 2), mimicking the well‐established relationship between $\dot{V}$O_2_ and work rate observed consistently in CE CPET. The potential value of this approach to TM CPET, *which uses only the actually measured data,* that is*, body mass, HR, and treadmill speed and incline*, is highlighted in Figure 5C, showing the very high correlation between the submaximal ΔVIM/ΔHR of TM CPET and ΔWR/ΔHR measured in CE CPET. As shown in Table 3, maturation‐ and sex‐related changes in TM CPET‐derived ΔVIM/ΔHR paralleled, as expected, the changes in CE ΔWR/ΔHR. Similarly, for exercise biomarkers expected to be relatively size independent, we found, as expected, little or no differences across our subgroups for CE CPET Δ$\dot{V}$O_2_/ΔWR and the parallel TM CPET Δ$\dot{V}$O_2_/ΔVIM.

We found strong correlations between peak $\dot{V}$O_2_ obtained from TM and CE CPET (Fig. 6A). Our data are consistent with previous studies demonstrating generally lower values for CE peak $\dot{V}$O_2_. For both TM and CE CPET, there was a strong correlation between Δ$\dot{V}$O_2_/ΔHR and peak $\dot{V}$O_2_ (Fig. 3B). This observation may be particularly useful in instances when a participant or patient does not meet standard criteria for peak $\dot{V}$O_2_, not an infrequent occurrence (Paridon et al., 2008). In these cases, investigators or clinicians might consider using Δ$\dot{V}$O_2_/ΔHR (a value not dependent on maximal effort) as a surrogate index for fitness.

Myers et al. (2017 and Kaminsky et al. (2017 recently published normative values for CPET in separate populations of adults using TM and CE. Although the investigators found generally lower peak $\dot{V}$O_2_ values in CE CPET, they were unable to identify a unique conversion factor that could eliminate differences between the two ergometer types across the age groups of their study. A number of investigators have compared TM and CE CPET in which participants performed both modalities (Jacobs and Sjödin, 1985; Turley and Wilmore, 1997; Basset and Boulay, 2000; Mitchell et al., 2010; Gordon et al., 2012; Itoh et al., 2013) and consistently higher TM CPET peak $\dot{V}$O_2_ has been observed. As noted, Turley and Wilmore (1997) studied both children and adults, and found that CE peak $\dot{V}$O_2_ in all groups was lower than TM to a small but consistent degree. There are a number of possible explanations for the higher peak $\dot{V}$O_2_ values in TM exercise, including the energy cost of maintaining an upright posture (Miles‐Chan et al., 2013; Júdice et al., 2016) and/or factors related to work efficiency, skeletal muscle mass, and activation that occur in TM but not CE CPET. Muscle mass clearly plays a role; for example, peak $\dot{V}$O_2_ is, as expected, smaller in upper body ergometry compared with TM or CE CPET (Drescher et al., 2015). It is noteworthy that we could not find significant changes in any of our submaximal slopes in the transition from walking to running on the treadmill, suggesting that the predominant component of energy costs of TM exercise is related to velocity, mass, and incline.

Using the strong relationships of LBM to both TM and CE peak $\dot{V}$O_2_ and Δ $\dot{V}$O_2_/ΔHR, we addressed the question of whether the two modalities reflected similar hierarchies in fitness among the participants. To do this, we used the linear regression relationship between LBM and both peak $\dot{V}$O_2_ and Δ$\dot{V}$O_2_/ΔHR to determine a predicted value for each participant based on LBM. We then correlated the TM and CE percent predicted value for each participant. As shown in Figures 3C and 6C, we found modest but significant correlations in fitness hierarchy for both submaximal Δ$\dot{V}$O_2_/ΔHR and peak $\dot{V}$O_2_. In summary, a participant in our study with a relatively high or low peak $\dot{V}$O_2_ would likely have respectively high or low Δ$\dot{V}$O_2_/ΔHR on both TM and CE modalities. However, the variability in our data also cautions that relative fitness ascertained by CPET biomarkers is not fully interchangeable between the two modalities.

Limitations: Due to the same order of exercise test modalities a sequence effect may influence the second test session. In this study supramaximal test was not performed. In children V'O_2_peak is more commonly used than in adults and supramaximal tests are equivocal. This study focused on submaximal values and novel TM protocol; thus, measuring peak or max values were presented as secondary end point.

In conclusion, our data reveal the effect of the two predominant modalities of laboratory exercise testing in children, adolescents, and young adults on submaximal and peak CPET results. Both modalities similarly reflected effects of body size on Δ$\dot{V}$O_2_/ΔHR, Δ$\dot{V}$E/Δ$\dot{V}$CO_2_, and peak $\dot{V}$O_2_. Results from the two modalities, however, are not interchangeable and may reflect the complexities of how external work, particularly on the treadmill, is transduced to physiologic responses such as $\dot{V}$O_2_ (Pandolf et al., 1977; Epstein et al., 1987; Hall et al., 2004) The reasons for using TM or CE exercise for assessment of exercise biomarkers in the clinic or in research ultimately depend on a variety of factors, including the skill and experience of the laboratory, available equipment, and perceived capabilities of the targeted participants or patients. We provide a novel approach for analyzing TM CPET data relying on actually measured HR, body mass, and the velocity and incline. This approach might prove useful in reanalyzing existing datasets where such measurements are available and in the future establishment of normative values for CPET testing in children and adolescents, where reliable datasets in large numbers of healthy participants are currently lacking.

## Conflict of Interest

The authors declare no conflicts of interest. The results of the study are presented clearly, honestly, and without fabrication, falsification, or inappropriate data manipulation.
