# The effect of test modality on dynamic exercise biomarkers in children, adolescents, and young adults

**DOI:** 10.14814/phy2.14231

**Published:** 2019-09-08

**Authors:** Ronen Bar‐Yoseph, Janos Porszasz, Shlomit Radom‐Aizik, Annamarie Stehli, Pearl Law, Dan M. Cooper

*Physiol Rep, 7 (17), 2019 e14178*.

https://doi.org/10.14814/phy2.14178

Dr. Kim D. Lu was inadvertently omitted from the authorship list. Dr. Lu was involved in all aspects of this manuscript including: development of project hypotheses and methods, data collection, analysis, and the writing and review of the final submission.

Kim D. Lu MD

Pediatric Exercise and Genomics Research Center

Department of Pediatrics, University of California, Irvine, School of Medicine
